# Determining the Optimal Harvesting Moment of Green Forage from *Guizotia abyssinica* Cultivated as a Catch Crop on Silage and Its Quality Form, Fresh or Wilted Green Material, in the Two Following Years

**DOI:** 10.3390/ani14172455

**Published:** 2024-08-23

**Authors:** Anna Szuba-Trznadel, Tomasz Hikawczuk, Anna Jama-Rodzeńska, Joanna Kamińska, Zlatko Svecnjak, Zygmunt Król, Bogusław Fuchs

**Affiliations:** 1 Department of Animal Nutrition and Feed Management, Wroclaw University of Environmental and Life Sciences, J. Chełmońskiego 38d, 51-630 Wrocław, Poland; anna.szuba-trznadel@upwr.edu.pl (A.S.-T.); boguslaw.fuchs@upwr.edu.pl (B.F.); 2 Statistical Analysis Centre, Wroclaw Medical University, K. Marcinkowskiego 1, 50-368 Wrocław, Poland; tomasz.hikawczuk@umw.edu.pl; 3 Institute of Agroecology and Plant Production, Wroclaw University of Environmental and Life Sciences, Pl. Grunwaldzki 24A, 50-363 Wrocław, Poland; 4 Department of Applied Mathematics, Faculty of Environmental Engineering and Geodesy, Wrocław University of Environmental and Life Sciences, 50-363 Wroclaw, Poland; joanna.kaminska@upwr.edu.pl; 5 Department of Filed Crops, Forage and Grasslands, Faculty of Agriculture, University of Zagreb, 10000 Zagreb, Croatia; svecnjak@agr.hr; 6 Saatbau Poland Sp. z o.o., Żytnia 1, 55-300 Środa Śląska, Poland; zygmunt.krol@saatbau.com

**Keywords:** *Guizotia abyssinica*, maturity, fermentation, nutrient composition, silage quality

## Abstract

**Simple Summary:**

*Guizotia abyssinica* is a plant cultivated mainly in Ethiopian and Indian climate conditions. Its seeds can be used to produce oil or as feed for ornamental birds. In recent years, interest has increased in its use as a catch crop between crops in European conditions. However, owing to its rapid growth rate, attention has also been paid to the possibility of ensiling this plant and using it to prepare feed for cattle after harvesting 58, 68, and 90 days after seed sowing. The results of this research indicate that collecting silage material on the 90th day after sowing allows for the preparation of very good-quality silage.

**Abstract:**

*Guizotia abyssinica* is currently being used for soil improvement; however, owing to its rapid growth and high productivity, it may have value as feed for ruminants, although this has not been well studied. Thus, this research aimed to evaluate the silage quality of *Guizotia abyssinica* grown during the short season (July–October) as a catch crop in northern Europe when harvested 58, 68, and 90 days after sowing (DAS) over two production years. Ensiled material was analyzed to compare silage quality for the three different DAS. Two factors were analyzed factorially in the experiment: the silage preparation year (2018 or 2019) and the form of the ensiled material (fresh or wilted). We used 36 replications, 18 for each variant of the experimental factor. Harvesting at 58 DAS resulted in unsatisfactory forage fermentability, even after wilting. At 68 DAS, silage quality was satisfactory, but the dry matter content before ensiling was below 20% for both fresh and wilted forage, indicating limitations for silage use without additional wilting for that DAS harvest time. Dry matter content and water-soluble carbohydrates consistently increased as harvest was delayed. Thus, the highest silage quality was obtained from forage harvested 90 DAS regardless of differences in dry matter content. Therefore, it is possible to prepare silage at lower temperatures when the wilting process is limited by environmental conditions.

## 1. Introduction

*Guizotia abyssinica* is an annual oilseed plant belonging to the Asteraceae family and the Guizotia genus [1]. This taxon comprises only six species, of which five are native to Ethiopia, and it can be cultivated as a seed and forage crop [2]. It was originally cultivated in Ethiopia and later introduced to India, becoming an important source of oil and animal feed [3,4,5,6], and is also cultivated in Sudan, Uganda, Zaire, Tanzania, Malawi, Zimbabwe, the West Indies, Nepal, Bangladesh, and Bhutan [7]. This plant is characterized by its high significance in sustainable food security in Ethiopia, where it is produced exclusively by smallholders [8]. Its most important part relates to noug seeds, an important source of protein, carbohydrates, vitamins, and fiber, all of which significantly contribute to the human diet. The oil of *Guizotia abyssinica* is also used to make soap and paint and as a lubricant and lighting fuel [9]. In Western European countries, *Guizotia abyssinica* seeds are valuable components in feed mixtures for ornamental birds [10,11]. Because of the high proportion of unsaturated fatty acids, mainly oleic and linoleic, and its lack of toxic substances, *Guizotia abyssinica* is also used in medicine in Ethiopia and India [11,12].

Depending on its growth rate and sowing date (from June to January of the following year), *Guizotia abyssinica* needs from 50 to 100 days to reach the flowering stage under Ethiopian conditions, and maturity can be reached from 120 to 180 days after sowing (DAS) [10]. Recently, this crop has been used as a catch crop in cultivation to enrich soil with organic matter in countries such as Germany, Austria, the Czech Republic, Croatia, and Brazil [11,12,13,14]. Owing to its rapid growth rate, it is possible to obtain a high amount of green matter, enabling the use of *Guizotia abyssinica* as feed for ruminants [6,14,15]. It may grow up to 2 m in height, and the whole plant can be used as green forage for sheep and goats (usually until the flowering stage); however, it is preferred in the form of silage for dairy cows and beef cattle [1,16]. Nega and Melaku [17] proved that supplementing hay with noug seed meal improves the dry matter (DM) intake, apparent digestibility coefficient, and body weight (BW) performance of Farta sheep, probably because of better availability and increased nutrient use.

In vitro studies have shown that *Guizotia abyssinica* affects the fermentation profile of bacteria in the rumen, consequently reducing methane emissions such as greenhouse gases [18]. In addition, Sun et al. [19] pointed to the possibility of reducing methane emissions by changing the proportion of fatty acids in the diet. Thus, *Guizotia abyssinica* could be used in catch cropping systems with other protein plants (for example, soybean). The highest soybean and *Guizotia abyssinica* green mass was obtained in a 75:25 planting ratio, respectively [20]. In addition, the highest total dry mass accumulation occurred up to 90 days after planting in the abovementioned ratio.

In Poland, attempts have been made to use *Guizotia abyssinica* as a catch crop to replace, for example, mustard, oats, and rye in crop rotations. Moreover, previous studies indicate that this rapidly plant grows at high temperatures and under water-deficit conditions [1,21]. This crop is characterized by a high genetic diversity, can be introduced under various climatic conditions, and is used as a fresh material for silage production [22].

This study aimed to evaluate the quality and nutritive value of silage made of wilted forage (at the level that was possible given weather conditions and stem water content) and fresh green forage in microsilos from plants cultivated under the climatic conditions of southwestern Poland gathered at 58, 68, and 90 DAS.

## 2. Materials and Methods

### 2.1. Establishing the Field Experiment

The experiment was carried out on a farm in the Opole voivodeship, Prudnik Municipality, Poland. The farm is in an area where the average annual air temperature is +8 °C, and precipitation is around 640 mm. The soil is classified as quality class III according to the Polish classification of soil quality [23]. The experiment was conducted on the same crops in 2018 and 2019. The previous crop was *Brassica napus var napus*, with a winter variety in 2018 and a spring variety in 2019. The experimental area was about 1000 m^2^. The crop was sown on 21.07.2018 and grown until 19.10.2018, and in the next year from 2.08.2019 to 31.10.2019 with a sowing rate of 9 kg ha^−1^. Only nitrogen fertilization was applied at the beginning of the field experiment at 100 kg ha^−1^, as described by Szuba-Trznadel et al. [21]. Seed material was obtained from the Saatbau Poland Company [24].

**Figure 1:**
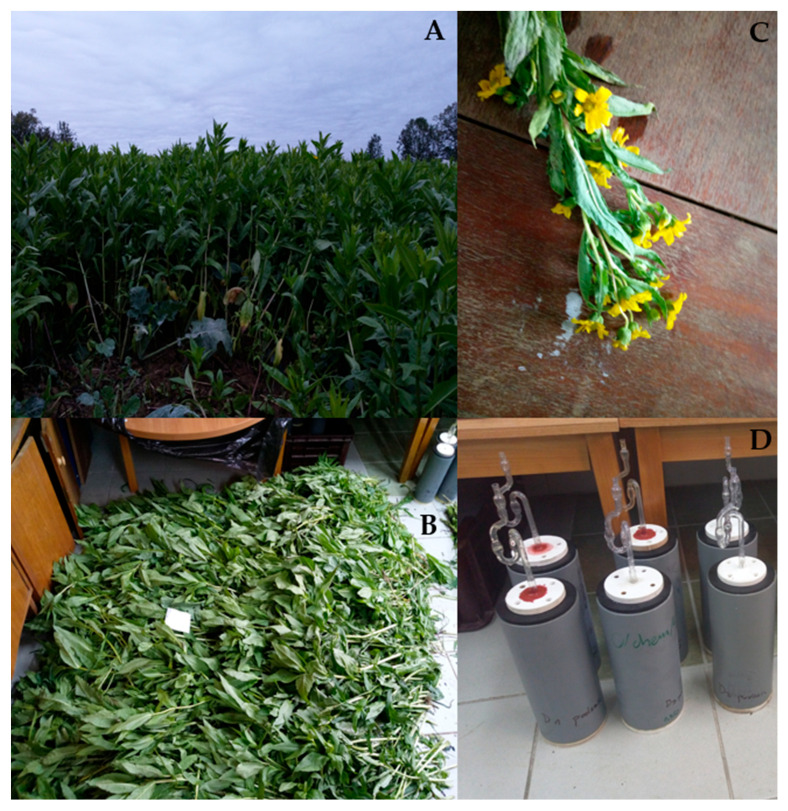
*Guizotia abyssinica* during vegetation and in a laboratory (**A**)—plant in a field. (**B**)—Fresh plant before the ensilage process. (**C**)—*Guizotia abyssinica* with flowers. (**D**)—Wilted *Guizotia abyssinica* in microsilos.

The crop was harvested 58 (early harvest), 68 (mid-harvest), and 90 (late harvest) DAS. From the entire field, 10 samples were representatively gathered from 10 squares 50 × 50 cm, as shown on Figure 1A. The material was collected with a sickle and cut at a height of 4 cm. Afterward, all fresh material from each square was mixed to obtain homogenous material to prepare silage; the same process was performed with wilted material gathered after the wilting process, as described by Szuba-Trznadel et al. [21].

Based on chemical analysis, laboratory-scale silage was created only from two harvest days, i.e., 68 and 90 DAS. The fresh material was cut into small pieces about 2 cm long and placed in airtight polyethylene microsilos with a capacity of 1500 cm^3^ (Figure 1 B–D). After the green forage was thoroughly compressed, the containers were immediately closed with a sterile rubber stopper. Owing to the presence of a fermentation tube (filled with glycerine), gas formed during the ensiling process could leave the microsilos, but air could not infiltrate them. Silage was created in two variants in thirty-six replicates from fresh or wilted material; the wilting process was performed under field conditions—the material was sundried for two days, and during that process, it was turned regularly to prevent uneven drying or decay in the plants. After 10 weeks of fermentation, the nutritive value of the silage was determined. In both years, quality was evaluated, taking into account the following parameters: buffer capacity [25], water-soluble carbohydrate value [26], and the fermentability coefficient according to the Weissbach equation [27]. Table 1 describes the design of the experiment (DOE) of our study (2 × 2).

### 2.2. Chemical Analysis

The nutrient concentration was determined in a laboratory at the Department of Animal Nutrition and Feed Science, Wroclaw University of Environmental and Life Sciences, Wrocław, Poland. The basic nutrient content in silage samples was determined using standard AOAC methods [28]. Before chemical analyses, silage samples were dried at 55 °C in dryers and then ground to a 1-millimeter-diameter particle size using a Fuchs Műhlen laboratory grinder, Type MM125H, Austria, 1974.

The DM of the laboratory samples was examined via the gravimetric method at 105 °C for four hours according to the Polish standard [29]. The chemical composition of the green forage was assessed according to AOAC International Official Methods of Analysis. Crude protein (CP) content was multiplied by nitrogen percentage (N %), as determined in the sample using a Kjeltec 2300 Foss Tecator apparatus (Foss A/S Company, Hillerød, Denmark; AOAC: 984.13) with a factor of (6.25). Crude ash (CA) was determined by combusting the sample in a muffle furnace (Czylok Company, Jastrzębie-Zdrój, Poland) at 550 °C for 24 h (AOAC: 942.05). Crude fat (ether extraction) (EE) was determined using the Soxhlet method, comprising extraction with ethyl ether (AOAC: 920.39A). Crude fiber (CF) was determined with the Hennenberg–Stohman method (AOAC: 978.10) using a Fibertec Foss Tecator apparatus for laboratory analysis (Foss A/S Company, Hillerød, Denmark). Mineralization was determined using a Mars 5 version 194A06 (CEM Corporation, Matthews, NC, USA) microwave mineralization system using HNO_3_.

Neutral detergent fiber (NDF) and acid detergent fiber (ADF) were determined with the Van Soest et al. method [30] using an adapter for an Ankom 200 Fiber Analyzer (Ankom Technology Corporation, New York, NY, USA). The gross energy (GE) of green forage was determined in a calorimetric bomb calorimeter (KL-11 “Mikado”, Precyzja-Bit Sp. z o.o., Bydgoszcz, Poland). Quality parameters were measured using a pH-meter lab (827 pH; Metrohm Company, Herisen, Switzerland), including silage fermentation acids, nitrogen–ammonia, and pH value [31]. The Lepper distillation method determined the amounts of acetic, butyric, lactic acid, and N-NH_3_ in the silage. The proportion of individual silage fermentation acids in the total acids and silage quality were evaluated using the Flieg–Zimmer score [32]. Volatile fatty acids were also determined using gas chromatography to compare the silage contents, except those mentioned above: propionic, isobutyric, valeric, isovaleric, and ethanol concentrations.

### 2.3. Statistical Analysis

The data from sampling on specific dates were initially prepared in Microsoft Office 365 Excel (version 2407, Microsoft Inc., Redmond, WA, USA). Then, all numerical data were evaluated using one-way (DAS) or two-way (year × wilted of fresh) ANOVA with Statistica version 13.3 (Tibco Software Inc., Palo Alto, CA, USA [33]). The results are presented as the mean value from 9 replications in each treatment (36 in total) for the one-way ANOVA and 18 replications for each sub-treatment for the two-way ANOVA analysis (2 × 2, Table 1). Data normality distribution was checked using the Shapiro–Wilk test. Homogeneity of variance was determined using Levene’s test. Differences in mean values between treatments were analyzed for significance (*p* < 0.05 or *p* < 0.01) using Tukey’s test. Dispersion of the data in treatments is presented using the standard error of the mean (SEM).

## 3. Results

The suitability of *Guizotia abyssinica* green forage for silage is presented in Table 1. In 2018, the harvested material was characterized by a significantly higher (*p* < 0.01) water-soluble carbohydrate (WSC) content at 90 DAS than at 68 DAS (an increase of over 30%). Compared with 2018, the WSC content was higher in the following (2019) growing season (Table 2).

Similarly to the previous growing season, the WSC content in 2019 increased with the later harvest and was significantly higher (*p* < 0.05) 90 DAS than the harvest three weeks earlier. However, an opposite response pattern was found for the buffer capacity (BC) of the silage material. In 2018, the buffer capacity decreased by 25% (*p* < 0.01) with harvest at 90 DAS (Table 2). This result was also found for 2019, where the decrease was ca. 36.5% (*p* < 0.01). At harvest 90 DAS, the lactic acid content was 26 g∙kg^−1^ DM compared with 41.1 g lactic acid kg^−1^ of DM at 68 DAS. In both growing seasons, the fermentability coefficient (FC) increased (*p* < 0.01) owing to the increased *Guizotia abyssinica* green forage DM. These increases between 68 and 90 DAS averaged 40% in 2018 and 53% in 2019 (Table 2).

**Figure 2:**
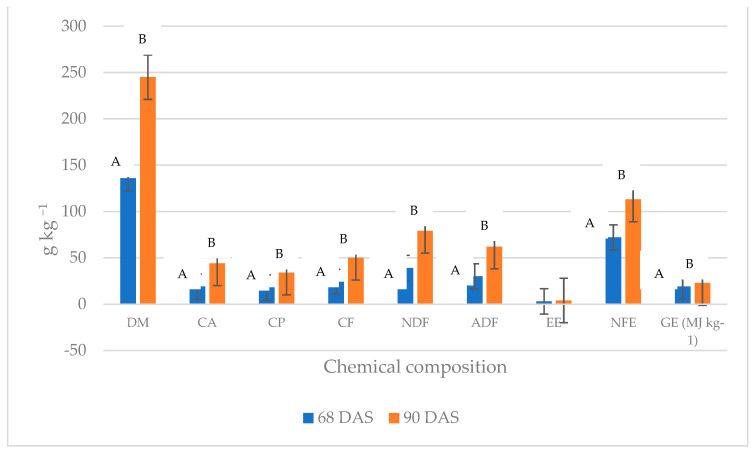
Comparison of chemical composition and gross energy level of silages prepared with fresh material of *Guizotia abyssinica* at 68 and 90 DAS (%) from both years (2018 and 2018) at Opole voivodeship, Poland. DM—dry matter; CA—crude ash; CP—crude protein; CF—crude fiber; NDF—neutral detergent fiber; ADF—acid detergent fiber; EE—ether extract; NFE—nitrogen-free extract; GE—gross energy. Significant differences marked within columns with different superscript capital letters indicate *p* ≤ 0.01. Values are the means of 18 replicates per year.

Figure 2 presents the chemical compositions of silages prepared with fresh materials from 2018 and 2019 (*n* = 36) gathered in two treatments (*n* = 18). A significant difference (*p* < 0.01) was found between 60 and 90 DAS in most of the chemical ingredients, except for EE (*p* > 0.05).

Table 3 presents the chemical composition of silage plant material harvested 68 DAS. Plant material field wilting before ensiling changed the values of the GE content and DM, CF, NDF, and ADF fraction parameters compared with the fresh material (*p* < 0.01). The EE concentration decreased (*p* < 0.01) because the material wilted before ensilage. The NFE concentration in the wilted material also decreased (Table 3, *p* < 0.01) compared with silage made from fresh material. Conversely, the wilting process increased the CA concentration (*p* < 0.05). There were no significant differences (*p* > 0.05) between fresh and wilted materials in the amount of CP in the ensiled silage variants made from *Guizotia abyssinica* (Table 3*)*.

A significantly higher (*p* < 0.01) plant material DM content in silage was found in 2019 than in 2018 (Table 3). Conversely, a significantly lower CP content was observed in 2019 than in 2018 (less than 23%). In addition, EE concentrations were significantly lower (*p* < 0.01) in 2019 than in 2018. There were no statistically significant differences between other nutrient concentrations in the years of study (Table 3, *p* > 0.05).

Table 4 presents the nutrient content in *Guizotia abyssinica* silage 90 DAS. In 2018, silage plant material had higher (*p* < 0.01) DM (more than 9%), CP (more than 8.5%), and EE (as much as more than 57%) content than in 2019. Consequently, the amount of CF and its fractions (ADF and NDF) in the plant material samples was higher (*p* < 0.01) in 2019 than in 2018 (about 23% in each nutrient). There were no significant differences (*p* > 0.05) in the EE or NFE levels (Table 4). Similarly, there were no significant differences in GE values between years. The wilting of green material in the field increased (*p* < 0.01) the levels of DM and all nutrients except EE and NFE. In those nutrients, there were no significant (*p* > 0.05) differences after the wilting process. The wilting process significantly (*p* < 0.01) decreased the amount of GE. An interaction between the year and silage material was observed for CP (*p* < 0.01) as well as a tendency in the case of EE (Table 4, *p* < 0.10).

This is demonstrated in Figure 2 by the non-parallel lines connecting the averages for the consecutive years. Thus, the desiccation effect of silage material on CP and EE content varies from year to year. Crude protein content in fresh and wilted silage differed significantly in 2018 (112.3 and 149.4 g·kg^−1^ of DM, respectively), while in 2019, it was very similar (117.3 and 122.4 g·kg^−1^ of DM, respectively, Figure 2). Thus, desiccation in 2019 had almost no effect on the CP content of silage, even though the same treatment increased the CP content by 37.1 g·kg^−1^ DM (33%) the year before. In 2019, the sum of precipitation was much lower than that in 2018, and therefore, additional drying was unnecessary.

**Figure 3:**
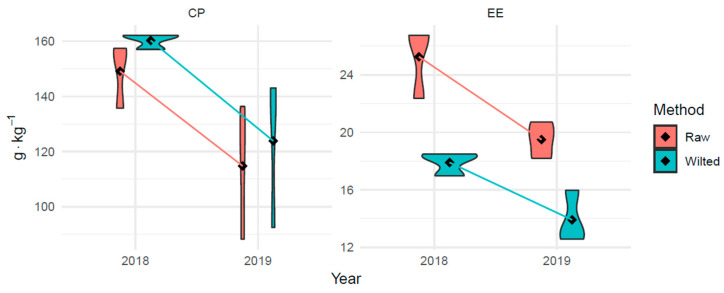
Violin plots with mean (black squares) and ANOVA Year*Silage interactions for plant material of *Guizotia abyssinica* for silage harvested 90 DAS in 2018 and 2019 at Opole voivodeship, Poland. CP—crude protein; EE—ether extract. Values are the means of 18 replicates per year. After the ensiling process to detect short-chain fatty acid concentrations, N-NH_3_ and the pH were determined (Table 5). No butyric acid was found in any silage. Considering that the acetic acid concentration was significantly higher (*p* < 0.01) in 2018, the wilting process significantly increased the lactic acid concentration (*p* < 0.01).

The opposite is true for EE; in 2018, the impact of plant material desiccation was smaller (the violin plots in Figure 2 are closer together) than in 2019 (violin plots are farther apart—a larger value difference). Thus, the gross energy in the resulting silage was significantly more influenced by drying the plant material in 2019. Higher values for these parameters (CP and EE) were obtained in a year with higher precipitation and air temperature; therefore, higher temperatures with moderate rainfall favor higher total protein and crude fat accumulation in plant material (Figure 3). These observations confirm the significant influence of growing conditions (weather) on the quality of plant material and the feedstock preparation method (fresh or wilted) used for silage production.

The opposite effect was observed in lactic acid (*p* < 0.01); higher levels of acetic acid also decreased lactic acid concentrations, which were significantly lower (*p* < 0.01) than those with other treatments given our one-way ANOVA results (Table 5). No significant differences (*p* > 0.05) were observed in different years considering N-NH_3_ concentrations and pH. Higher N-NH_3_ concentrations and, consequently, pH levels were determined (*p* < 0.01) in silage from fresh material. Silage in 2019 scored significantly higher (*p* < 0.01) on the Flieg–Zimmer scale than silage prepared in 2018. Silage from wilted material scored higher on the Fileg–Zimmer scale (*p* < 0.01) than silage prepared with fresh material. All silage at over 68 days obtained over 90 points on the scale, qualifying it as very good (Table 5).

In silages from material collected 90 DAS, no butyric acid was found in any one sample (Table 6). Considering acetic and lactic acid concentrations, no significant differences between years and the type of material were detected (*p* > 0.05). Significantly lower (*p* < 0.05) N-NH_3_ concentrations were found in 2018 than in 2019; a lower concentration (*p* < 0.01) of this chemical compound was observed in silage from wilted material (Table 6). The year did not significantly influence differences in values (*p* > 0.05), with fresh material in the ensilage process significantly increasing its pH (*p* < 0.05). The wilting of green material collected 90 DAS did not influence the silage quality (*p* > 0.05). All silage scored 97.7 to 98.0 points out of 100, emphasizing its very good quality (Table 6).

**Figure 4:**
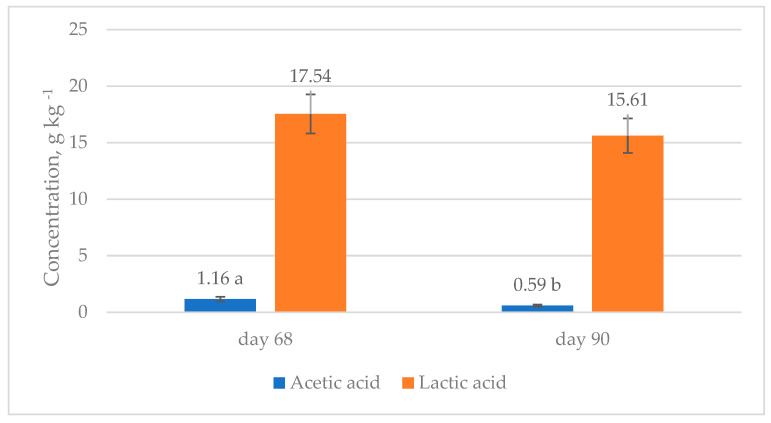
Concentrations of most abundant short-chain fatty acids in silage from fresh material prepared from plants of *Guizotia abyssinica* gathered at 68 and 90 DAS in 2018 and 2019 at Opole voivodeship, Poland. Letters a and b describe significant differences at *p* < 0.05. Values are the means of 18 replicates per year.

Figure 4 presents the most abundant SCFA levels in silage prepared on two different DAS (68 and 90). A significant difference was observed in the acetic acid concentrations (*p* < 0.05) but no significant differences were observed (*p* > 0.05) in the lactic acid concentrations (Figure 3).

Table 7 presents an evaluation of silage prepared 68 days after the sowing of *Guizotia abyssinica,* based on the fatty acid contents determined using the chromatography method. Considering the sowing year, a significantly higher (*p* < 0.05) lactic acid concentration was determined for 2018. The acetic and valeric acid amounts were significantly higher in 2019 (Table 7, *p* < 0.01). Conversely, significantly higher (*p* < 0.01) propionic acid and ethanol concentrations were observed for 2018. On the other hand, the isobutyric acid and isovaleric acid contents were below the detection limit, and butyric acid showed no differences (*p* > 0.05) between the years observed (Table 7).

Wilted and fresh silage showed no significant differences (*p* > 0.05) after the ensilage process for lactic, propionic, isobutyric, butyric, and isovaleric acid. The wilting of silage material resulted in a greater decrease (*p* < 0.01) in the concentration of acetic acid than the use of fresh material (by almost 49%). A significant difference (*p* < 0.05) was found in the ethanol concentration (wilted silage material characterized by 78% less ethanol content). In silage from wilted material, higher (*p* < 0.01) valeric acid and N-NH_3_ concentrations were observed (Table 7).

Table 8 presents an evaluation of silage made 90 DAS from *Guizotia abyssinica* based on fatty acid content determined via the chromatography method. There were no significant differences (*p* > 0.05) between the years considering lactic, isobutyric, butyric, isovaleric acid, and ethanol concentrations (Table 8). There were higher (*p* < 0.05) acetic acid and N-NH_3_ concentrations in 2019. The amount of propionic acid was also significantly higher (*p* < 0.01) in 2018. Conversely, (*p* < 0.01) valeric acid concentrations in 2019 were higher (Table 8).

Our analysis of ensiled silage material showed no significant differences (*p* > 0.05) in lactic, isobutyric, butyric, isovaleric, and valeric acid concentrations (Table 8). Acetic acid concentrations were higher (*p* < 0.05) in silage from wilted material. Moreover, ethanol and N-NH_3_ were higher (*p* < 0.01) in silage from wilted material. Conversely, the propionic acid concentrations were lower than those in fresh silage material (Table 8).

## 4. Discussion

The stability of silage depends on the ensiled material, and this is an important factor in determining its quality and nutritional value. This study evaluated the effect of using certain types of ensiled *Guizotia abyssinica* material on the quality of silage depending on the date of forage harvesting. An increased average annual temperature has led to interest in cultivating field crops from the tropical zone [34,35,36,37]. Under European conditions, *Guizotia abyssinica* is mostly cultivated as a catch crop rather than a main crop [5]. However, the literature discusses its use as livestock feed: green forage is mainly used for goat and sheep feed. Because of the high essential oil content in green forage, cattle are reluctant to eat it, and therefore, attempts have been made to ensile it [38].

Forage preservation occurs under anaerobic conditions, wherein microorganisms use fermentable forage sugars to produce organic acids, mainly lactic acid [39]. Some techniques can preserve green fodder for future use, and the ensiling process has become a common practice for Polish farmers in recent years. In our experiment, we used airtight polyethylene microsilos with fresh and wilted material ensiling an uncommon plant in Poland. Ojeda et al. [40] found that wilting was the most effective treatment, providing the best rates and ammonia content. Marsh [41] found only positive aspects to silage wilting, and Ojeda et al. [42] found that when mulberry leaves are dried in the sun, they lose water faster, so proteases should be inactivated quickly, reducing their action during fermentation. In our study, wilted material was also more beneficial and improved the silage quality. Lyimo et al. [43] found that wilted grass silage showed higher DM, CP, ash, and WSC contents but lower NDF content than fresh grass silage. Our results also agree with Mtengeti et al. [44], who found improvements after wilting forage grasses, and Nussio [45], who found not only higher DM and WSC values but also a reduction in unwanted fermentation.

The WSC content consistently increased with later green forage harvesting; consequently, the highest plant material quality for silage was obtained at 90 DAS (FC = 96–111) and the lowest was obtained from crops harvested at 58 DAS (FC = 26–37, Table 2). The fermentability of green forage below 45 DAS is considered a limiting value; above this value, the material is assumed to ensile well [46]. Thus, good fresh material for ensiling could be harvested at 68 and 90 DAS. Ali and Tahir [47] stated that WSCs are a key source of fermentation products; in the anaerobic environment, these compounds are converted into organic acids, including lactic acid, with the presence of lactic acid bacteria, affecting the microbial stability of silage by decreasing the pH. Our WSC content results are promising in considering *Guizotia abyssinica* for catch cropping with soybean [35] and other protein-feed crops, like alfalfa [34]. However, by selecting the right *Guizotia abyssinica* genotype, it is possible to obtain plants with similar growth rates to soybeans. Recommended proportions of *Guizotia abyssinica* and soybeans are 50:50 or 75:25, which could reduce the competition between these plants for the nutrients and solar energy necessary for photosynthesis [20].

The average DM content of the silage variants (Table 3 and Table 4) increased with later forage harvesting. In the middle date of harvest (68 DAS), DM ranged from 13 to 14% (without wilting) and from 17 to 19% (after wilting). The DM values in our study were low; this may require further research using a range of drying-process periods and the determination of an appropriate method. These changes are prompted by the anatomical structure of the stem (stiff, thick, and empty inside). Additionally, fermentation in silos can produce volatile compounds that reduce DM in ensiled material [48]. Mtengeti and Urio [44] noted that DM loss was higher in non-wilted grass silage than in wilted grass silage. This may be related to the lower moisture content in wilted grass, which increases the available grass DM retained during fermentation.

Statistical differences were noted between the variants with and without drying (*p* < 0.01) and the years of study (*p* < 0.01). In the last harvest term (90 DAS), statistically significant differences were observed (*p* < 0.01) when the DM content ranged from 250 g∙kg^−1^ (in 2019) to 270 g∙kg^−1^ (in 2018). The optimal DM content range for ensiling corn is 300–350 g∙kg^−1^, 250–300 g∙kg^−1^ for GPS, and 350 g∙kg^−1^ for sunflowers (there are no data in the nutritional value tables regarding silage from *Guizotia abyssinica*) [49,50,51]. Silage made from material harvested 90 DAS was characterized by lower DM values than recommended. The literature states that fresh material should not be ensiled below 250 g kg^−1^ of DM, as *Clostridium* bacteria are a potential danger, producing undesirable butyric acid [52]. Despite the low DM content, there was no butyric acid in our study; this may be connected to the maintenance of anaerobic conditions and the high WSC content obtained in very good-quality silage, receiving a total score above 85 on the Flieg–Zimmer scale.

The CP concentrations in silage (Table 3) were similar on the second harvest date (68 DAS) and ranged from 132 to 142 g∙kg^−1^ of DM. The higher the DM content, the lower the CP content in the analyzed silage (Table 3 and Table 4). The protein level also depended on the study year; in 2018, it was significantly higher than the value determined in 2019 (*p* < 0.01) (Table 3 and Table 4). *Guizotia abyssinica* has higher CP levels at 90 DAS than corn silage at the waxy maturity phase [34]. A statistically higher EE content in silage was noted in 2018 than in 2019 (*p* < 0.01) (Table 3 and Table 4). The average amount of EE was significantly (*p* < 0.01) lower in silage made from dried material than fresh material (*p* <0.01) (Table 3) owing to the evaporation of this kind of nutrient during the wilting process. In turn, more CF was determined in the dried material than in the fresh material (*p* < 0.01) (Table 3). The NDF content in silage may be reduced through the acid hydrolysis of hemicelluloses [53,54], increasing soluble substrate availability. Rezende et al. [55] showed greater reductions in NDF in silage using rehydrated corn grains than in water silage obtained at 90 DAS, characterized by a higher DM content and a significantly lower GE value than those prepared with silage material gathered at 68 DAS.

Anaerobic fermentation produces organic compounds (lactic acids, acetic acids, and alcohol). Lactic acid is the predominant fermentation product, maintaining minimal protein breakdown and degradation. In our study, this was the dominant acid, especially in fresh material in 2018. Low-fermentation-quality silage is connected to poor preservation; it is unpalatable to animals and interferes with consumption [56].

The wilted material was characterized by significantly higher lactic acid and nitrogen–ammonia (NH_3_N) (Table 5). The amount of acetic acid is related to DM, and low acetic acid content can be obtained in silage with high DM content. Statistically, the lowest acetic acid values were noted in 2019 (*p* < 0.01). Gerlach et al. [57] found that an acetic acid DM concentration of 17.3–60 gkg^−1^ decreases DM intake by 5.6 g in silage for each additional gram of acetic acid per 100 kg of BW. This observation was confirmed in a study by Han et al. [58]. The literature indicates that pH and DM correlate: the higher the DM content, the higher the pH value. Weiss et al. [59] found that properly protecting the fermentation process in transit silos, prisms, etc., affects the fermentation efficiency and quality of the final corn silage product. Postponing this process by 24 h can result in access to more air, altering the fermentation and reducing the production of lactic acid in the first ensiling stage, thus affecting oxidative stability and indirectly increasing pH. The low NH_3_N content in wilted material can be explained by proteolysis not taking place during fermentation [43].

Good silage should have a pH of 4.2. This guarantees high lactic, acetic, and propionic acid contents, reducing the number of yeasts, mycotoxins, and (potentially) pathogenic bacteria in ruminant microflora [60,61].

In our study, a higher pH was observed in the fresh material than in the wilted material, contrary to the study by Lyuimo et al. [43], where wilted silage was characterized by higher pH, lactic acid, and acetic acid but lower butyric acid than non-moistened grass silage. Keles et al. [62] also found higher pH in wilted grasses. The lactic-to-acetic ratio is a good indicator of silage fermentation efficiency. The ratio of these acids can be no less than 3:1, and higher is better, as seen in our study. The pH of the wilted grasses was related to the high WSC concentrations in the wilted herbs, increasing the subsequent fermentation quality of the silage [63,64]. None of the silage contained butyric acid, similar to the results of a study by Lyuimo et al. [43]. This can be explained by lower water activity in the wilted herbs [65], creating more inhibitory conditions for microbial fermentation [66]. This is a typical fermentation trait of *Clostridium* bacteria. These bacteria indicate silage quality and are very sensitive to water availability, requiring wet conditions for active development [67]. Organic acid content and excessive nitrogen–ammonia promote biogenic amine formation [68]. Our analysis of N-NH_3_ content in the total nitrogen showed this compound to be at a good level. According to Flynn [68], NH_3_-N levels in good silage should not exceed 10% DM. Additionally, silage made from fresh material gathered 90 days after sowing scores the same number of points as silage derived from the wilting process; this suggests that 90 DAS and a longer vegetation period can yield very good-quality silage using fresh green material without the wilting process.

## 5. Conclusions

When high-quality forage resources are in short supply, *Guizotia abyssinica* silage could be a promising nutritional supplement for ruminant diets. Considering WSC and BC values, we conclude that *Guizotia abyssinica* is suitable for ensiling and can be a valuable silage material. The correct harvest date for this crop is the key to obtaining valuable forage. Silage material gathered at 58 DAS does not provide satisfactory animal silage (45/100 points). In our study, the most beneficial harvest date for this plant was 90 DAS (minimally, 97/100 points), even in silage prepared with fresh material under less favorable environmental conditions. Additionally, increasing the DM concentration in green fodder during the vegetation period enables an ensiling process without wilting this plant in the field and can even shorten this period in unfavorable weather conditions. Precisely determining the amount that can be offered to animals will be the next step in introducing this feed to ruminant diets.

## Figures and Tables

**Table 1:** Design of experiment and data collection for two experimental factors tested on *Guizotia abyssinica* at Opole voivodeship, Poland.

Year (Previous Crop)	Material for Silage	
	Fresh	Wilted
2018 (winter rape*)*	*n* = 9 (1–9)	*n* = 9 (10–18)
2019 (spring rape)	*n* = 9 (19–27)	*n* = 9 (28–36)

**Table 2:** The suitability of green forage for silage tested on *Guizotia abyssinica* in 2018 and 2019 at Opole voivodeship, Poland.

Specification	DAS			*p-*Value
	58	68	90	
2018				
WSC (g kg^−1^ DM)	120 ^C^ ± 7.3	214 ^B^ ± 11.4	281 ^A^ ± 6.2	0.000
BC (g lactic acid kg^−1^ DM)	60 ^A^ ± 2.7	40 ^A^ ± 3.11	30 ^B^ ± 2.7	0.000
FC (DM (%)+ 8 WSC/BC)	25.70 ^C^ ± 5.00	56.10 ^B^ ± 7.26	96.03 ^A^ ± 4.45	0.000
2019				
WSC (g kg^−1^ DM)	161.2 ^Aa^ ± 6.1	225.2 ^b^ ± 5.7	292.0 ^B^ ± 5.1	0.000
BC (g lactic acid kg^−1^ DM)	47.95 ± 4.5	41.14 ^A^ ± 2.3	26.14 ^B^ ± 2.5	0.000
FC (DM (%)+ 8 WSC/BC)	37.36 ^A^ ± 5.3	57.99 ^B^ ± 4.00	111.06 ^C^ ± 3.8	0.000

WSC—water-soluble carbohydrate; BC—buffer capacity; FC—fermentability coefficient. Significant differences marked within rows with different capital letters indicate *p* ≤ 0.01; lowercase letters indicate *p* ≤ 0.05. Values are the means of 18 replicates per year.

**Table 3:** Chemical composition of plant material of *Guizotia abyssinica* for silage harvested at 68 DAS in 2018 and 2019 at Opole voivodeship, Poland.

Treatment	DM	CP	CF	EE	CA	NFE	NDF	ADF	GE
	%	g·kg^−1^ DM							MJ·kg^−1^ DM
Silage									
Fresh 2018	12.71 ^Bb^	149.13	167.74 ^Bb^	25.26 ^Aa^	144.91	512.95 ^ab^	271.93 ^Bc^	211.25 ^Cb^	18.77 ^Aa^
Wilted 2018	17.07 ^Aa^	160.27	224.35 ^ab^	17.90 ^Bbc^	158.15	439.32 ^b^	361.98 ^ab^	309.16 ^ABa^	19.77 ^Bb^
Fresh 2019	14.42 ^Bb^	114.77	182.82 ^Bb^	19.49 ^b^	141.24	541.67 ^a^	300.29 ^bc^	229.14 ^BCb^	18.15 ^Bc^
Wilted 2019	19.33 ^Aa^	123.84	244.04 ^Aa^	13.91 ^Bc^	153.98	464.23 ^ab^	398.12 ^Aa^	334.04 ^Aa^	20.06 ^Aa^
*p*-value	0.000	0.064	0.001	0.000	0.141	0.026	0.003	0.001	0.000
*SEM*	0.794	7.311	10.062	1.300	2.964	14.816	16.607	16.947	0.238
Year									
2018	14.89 ^B^	154.70 ^A^	196.05	21.58 ^A^	151.53	476.14	316.95	260.20	19.27
2019	16.87 ^A^	119.30 ^B^	213.43	16.70 ^B^	147.61	502.95	349.20	281.59	19.11
Silage material									
Fresh	13.56 ^B^	131.95	175.28 ^B^	22.38 ^A^	143.08 ^b^	527.31 ^A^	286.11 ^B^	220.20 ^B^	18.46 ^B^
Wilted	18.20 ^A^	142.06	234.20 ^A^	15.90 ^B^	156.06 ^a^	451.78 ^B^	380.05 ^A^	321.60 ^A^	19.91 ^A^
*p*-value									
Year	0.006	0.013	0.095	0.001	0.458	0.219	0.091	0.201	0.259
Silage material	0.000	0.391	0.000	0.000	0.033	0.006	0.001	0.000	0.000
Y*S	0.619	0.928	0.808	0.397	0.962	0.927	0.823	0.826	0.009

DM—dry matter; CP—crude protein; CF—crude fiber; EE—ether extract; CA—crude ash; NFE—nitrogen-free extract; NDF—neutral detergent fiber; ADF—acid detergent fiber; GE—gross energy. Significant differences marked within columns with different superscript capital letters indicate *p* ≤ 0.01; lowercase letters indicate *p* ≤ 0.05. Values are the means of 18 replicates per year.

**Table 4:** Chemical composition of plant material of *Guizotia abyssinica* for silage harvested 90 DAS in 2018 and 2019 at Opole voivodeship, Poland.

Treatment	DM	CP	CF	EE	CA	NFE	NDF	ADF	GE
	%	g·kg ^−1^ DM							MJ·kg^−1^ DM
Silage									
Fresh 2018	25.73 ^AB^	112.33 ^Bc^	163.39 ^Bc^	25.84 ^A^	155.08 ^Aa^	477.20	252.51 ^Cd^	184.19 ^B^	22.85 ^b^
Wilted 2018	28.03 ^A^	149.41 ^Aa^	210.25 ^ab^	24.92 ^A^	221.24 ^bc^	460.35	305.82 ^Bc^	254.51 ^C^	24.18 ^a^
Fresh 2019	23.33 ^B^	117.27 ^Bbc^	201.08 ^b^	19.43 ^B^	138.04 ^Bc^	458.59	339.04 ^Aa^	250.58 ^B^	22.79 ^b^
Wilted 2019	25.55 ^AB^	122.38 ^Bb^	239.30 ^Aa^	22.15 ^B^	208.52 ^ab^	493.24	382.54 ^Bb^	319.40 ^A^	24.11 ^a^
*p-*value	0.003	0.000	0.000	0.000	0.005	0.572	0.000	0.000	0.003
SEM	0.555	4.397	8.788	2.252	11.882	9.252	14.566	14.856	0.224
Year									
2018	26.88 ^A^	130.87 ^A^	186.82 ^B^	25.38 ^A^	188.16	468.77	279.16 ^B^	219.35 ^B^	23.51
2019	24.44 ^B^	119.83 ^B^	220.19 ^A^	20.79 ^B^	173.28	465.91	360.79 ^A^	284.99 ^A^	23.45
Silage material									
Fresh	24.53 ^B^	114.80 ^B^	182.24 ^B^	22.63	146.56 ^B^	465.45	295.77 ^B^	217.39 ^B^	22.82 ^B^
Wilted	26.79 ^A^	135.89 ^A^	224.77 ^A^	23.54	214.88 ^A^	469.24	344.18 ^A^	286.96 ^A^	24.15 ^A^
*p-*value									
Year	0.002	0.000	0.002	0.000	0.280	0.885	0.000	0.000	0.799
Silage material	0.004	0.000	0.000	0.327	0.001	0.848	0.000	0.000	0.000
Y*S	0.947	0.000	0.584	0.069	0.870	0.314	0.450	0.931	0.993

DM—dry matter; CP—crude protein; CF—crude fiber; EE—ether extract; CA—crude ash; NFE—nitrogen-free extract; NDF—neutral detergent fiber; ADF—acid detergent fiber; GE—gross energy; Significant differences marked within columns with different capital letters indicate *p* ≤ 0.01; lowercase letters indicate *p* ≤ 0.05. Values are the means of 18 replicates per year.

**Table 5:** Silage evaluation of *Guizotia abyssinica* via evaluated using the Lepper distillation and the Flieg–Zimmer scale on 68 DAS in 2018 and 2019 at Opole voivodeship, Poland.

Treatment	Share of Acids (%)			N-NH_3_ %N_total_	pH	Silage Quality according to Flieg–Zimmer Scale	
	Acetic acid	Butyric acid	Lactic acid			Score (Points) *	Quality **
Silage							
Fresh 2018	19.23 ^B^	0.00	80.77 ^A^	0.61 ^A^	4.18 ^a^	89.5 ^B^	very good
Wilted 2018	28.47 ^A^	0.00	71.53 ^B^	0.29 ^B^	3.91 ^Bb^	97.3 ^A^	very good
Fresh 2019	17.50 ^B^	0.00	82.50 ^A^	0.63 ^A^	4.39 ^Aa^	98.0 ^A^	very good
Wilted 2019	18.55 ^B^	0.00	81.45 ^A^	0.26 ^B^	3.91 ^Bb^	98.0 ^A^	very good
*p-*value	0.001	-	0.001	0.000	0.000	0.000	-
SEM	1.417	-	1.417	0.055	0.064	1.109	-
Year							
2018	23.9 ^A^	0.0	76.21 ^B^	0.45	4.05	93.4 ^B^	very good
2019	18.0 ^B^	0.0	82.02 ^A^	0.44	4.15	98.0 ^A^	very good
Silage material							
Fresh	18.37 ^B^	0.00	81.63 ^A^	0.62 ^A^	4.29 ^A^	93.8 ^B^	very good
Wilted	23.51 ^A^	0.00	76.49 ^B^	0.27 ^B^	3.91 ^B^	97.7 ^A^	very good
*p*-value							
Year	0.001	-	0.001	0.859	0.074	0.000	-
Silage material	0.003	-	0.003	0.000	0.000	0.000	-
Year*Silage material	0.009	-	0.009	0.520	0.064	0.000	-

* Number of scores according to Flieg–Zimmer scale; ** evaluation according to Flieg–Zimmer scale. Significant differences marked within columns with different capital letters indicate *p* ≤ 0.01; lowercase letters indicate *p* ≤ 0.05. Values are the means of 18 replicates per year.

**Table 6:** Silage evaluation of *Guizotia abyssinica* using the Lepper distillation and the Flieg–Zimmer scale on 90 DAS in 2018 and 2019 at Opole voivodeship, Poland.

Treatment	Share of Acids (%)			N-NH_3_ %N_total_	pH	Silage Quality according to Flieg–Zimmer Scale	
	Acetic Acid	Butyric Acid	Lactic Acid			Score (Points) *	Quality **
Silage							
Fresh 2018	16.2 ^a^	0.00	83.9	0.42 ^b^	4.12 ^b^	98.00	very good
Wilted 2018	18.8 ^ab^	0.00	81.8	0.61 ^Aa^	4.18 ^ab^	97.33	very good
Fresh 2019	18.0 ^ab^	0.00	82.5	0.63 ^Aa^	4.39 ^a^	98.00	very good
Wilted 2019	19.3 ^b^	0.00	80.7	0.19 ^Bc^	4.13 ^b^	98.00	very good
*p-*value	0.027	-	0.129	0.000	0.019	0.441	-
SEM	0.443	-	0.494	0.056	0.040	0.167	-
Year							
2018	17.52	0.00	82.81	0.52 ^a^	4.15	97.7	very good
2019	18.71	0.00	81.62	0.41 ^b^	4.26	98.0	very good
Silage material							
Fresh	17.73	0.00	82.28	0.62 ^A^	4.29 ^a^	97.7	very good
Wilted	18.40	0.00	82.13	0.31 ^B^	4.13 ^b^	98.0	very good
Year	0.087		0.183	0.028	0.066	0.347	-
Silage material	0.295		0.868	0.000	0.015	0.347	-
Year*Silage material	0.011		0.047	0.013	0.089	0.347	-

* Number of scores according to Flieg–Zimmer scale; ** evaluation according to Flieg–Zimmer scale. Significant differences marked within columns with different capital letters indicate *p* ≤ 0.01; lowercase letters indicate *p* ≤ 0.05. Values are the means of 18 replicates per year.

**Table 7:** Evaluation of the silage quality of *Guizotia abyssinica* via the chromatographic method (68 DAS) in 2018 and 2019 at Opole voivodeship, Poland.

Treatment	Lactic Acid	Acetic Acid	Propionic Acid	Ethanol	Butyric Acid	Valeric Acid	N-NH_3_ %N_total_
	g kg^−1^						
Silage							
Fresh 2018	20.353	0.847 ^B^	0.027 ^ab^	1.602 ^ab^	0.227	0.006 ^B^	0.051 ^Aa^
Wilted 2018	20.695	0.775 ^B^	0.030 ^Aa^	2.329 ^a^	0.004	0.001 ^B^	0.027 ^BCb^
Fresh 2019	14.942	0.713 ^B^	0.013 ^Bc^	0.604 ^a^	0.016	0.037 ^A^	0.046 ^ABa^
Wilted 2019	14.379	1.547 ^A^	0.017 ^bc^	1.391 ^ab^	0.009	0.010 ^B^	0.019^Cb^
*p-*value	0.147	0.000	0.006	0.016	0.300	0.000	0.000
SEM	1.294	0.107	0.002	0.220	0.048	0.004	0.004
Year							
2018	20.524 ^a^	0.811 ^B^	0.028 ^A^	1.965 ^A^	0.115	0.004 ^B^	0.039
2019	14.660 ^b^	1.130 ^A^	0.015 ^B^	0.998 ^B^	0.013	0.024 ^A^	0.032
Silage material							
Fresh	17.537	1.161 ^A^	0.023	1.860 ^a^	0.007	0.006 ^B^	0.023 ^B^
Wilted	17.647	0.780 ^B^	0.020	1.103 ^b^	0.121	0.022 ^A^	0.049 ^A^
*p-*value							
Year	0.029	0.005	0.000	0.008	0.288	0.000	0.117
Silage material	0.961	0.002	0.291	0.026	0.239	0.000	0.000
Year*Silage material	0.843	0.001	0.745	0.916	0.267	0.001	0.661

Significant differences marked within columns with different capital letters indicate *p* ≤ 0.01; lowercase letters indicate *p* ≤ 0.05. Values are the means of 18 replicates per year.

**Table 8:** Evaluation of silage quality of *Guizotia abyssinica* via the chromatographic method (90 DAS) in 2018 and 2019 at Opole voivodeship, Poland.

Treatment	Lactic Acid	Acetic Acid	Propionic Acid	Ethanol	Butyric Acid	Valeric Acid	N-NH_3_ %N_total_
	g kg^−1^						
Silage							
Fresh 2018	17.64	0.75 ^a^	0.09 ^A^	0.11 ^b^	0.01	0.02 ^ab^	0.04 ^a^
Wilted 2018	18.69	0.81 ^a^	0.03 ^B^	1.60 ^a^	0.05	0.01 ^b^	0.05 ^Aa^
Fresh 2019	13.58	0.43 ^b^	0.02 ^B^	0.00 ^b^	0.03	0.05 ^a^	0.02 ^Bb^
Wilted 2019	15.61	0.75 ^a^	0.01 ^B^	0.60 ^ab^	0.02	0.04 ^a^	0.05 ^Aa^
*p*-value	0.237	0.010	0.000	0.011	0.168	0.012	0.001
SEM	0.941	0.053	0.009	0.223	0.008	0.006	0.004
Year							
2018	18.164	0.783 ^a^	0.057 ^A^	0.858	0.028	0.013 ^B^	0.043 ^a^
2019	14.594	0.586 ^b^	0.018 ^B^	0.302	0.022	0.043 ^A^	0.032 ^b^
Silage material							
Fresh	15.611	0.589 ^b^	0.055 ^A^	0.057 ^B^	0.017	0.035	0.026 ^B^
Wilted	17.147	0.780 ^a^	0.020 ^B^	1.103 ^A^	0.034	0.022	0.049 ^A^
*p*-value							
Year	0.071	0.015	0.000	0.074	0.657	0.003	0.019
Silage material	0.397	0.017	0.000	0.005	0.248	0.099	0.000
Year*Silage material	0.782	0.075	0.003	0.141	0.061	0.918	0.135

Significant differences marked within columns with different capital letters indicate *p* ≤ 0.01; lowercase letters indicate *p* ≤ 0.05. Values are the means of 18 replicates per year.

## Data Availability

The raw data supporting the conclusions of this article will be made available by the authors on request.
